# Age-related injury patterns resulting from knife violence in an urban population

**DOI:** 10.1038/s41598-022-17768-x

**Published:** 2022-09-26

**Authors:** P. Vulliamy, K. Hancorn, S. Glasgow, A. West, R. A. Davenport, K. Brohi, M. P. Griffiths

**Affiliations:** 1 Centre for Trauma Sciences, Blizard Institute, Queen Mary University of London, London, E1 2AT UK; 2 The Royal London Hospital Major Trauma Centre, Barts Health NHS Trust, Whitechapel, E1 1FR UK

**Keywords:** Epidemiology, Trauma

## Abstract

Interpersonal violence involving knives is a major public health problem. The majority of patients are young people in urban areas, but little is known about age-specific patterns of injury and recent trends in injury characteristics. We performed a retrospective cohort study of all patients presenting to an urban major trauma centre with stab injuries resulting from assault between 2012 and 2018. A total of 3583 patients were included. Young people (age under 25) were more likely to have sustained multiple stab wounds compared to older people (43% vs 35%, *p* < 0.001) and had significantly higher rates of stab injuries involving the lower limbs, groin and buttocks. The annual number of injuries increased steadily during the study period in patients aged under 25 (r^2^ = 0.82, *p* = 0.005) and those over 25 (r^2^ = 0.95, *p* < 0.001). Over time, limb and junctional injuries accounted for an increasing proportion of stab wounds in young people, overtaking torso injuries as most common pattern of injury by the end of the study period. These findings illustrate the influence of age on injury patterns resulting from knife violence, and support the expansion of outreach initiatives promoting bystander-delivered haemorrhage control of extremity wounds.

## Introduction

Violence involving knives has been highlighted by the World Health Organisation as a pressing public health issue^1^, and interpersonal violence remains one of the leading causes of mortality among young people worldwide^2^. In the UK, the number of stabbings has increased significantly in many areas over the past decade, despite widespread media attention and numerous preventative initiatives from both local and national authorities^3,4^. In addition to the immediate physical harms, knife violence has profound detrimental effects on the psychological and social wellbeing of both affected individuals and the wider community^5^. Young people account for the majority of victims of knife violence in urban populations and are at particularly high risk of long-term psychosocial problems following exposure to violence^6–8^. Targeted initiatives to reduce the burden of interpersonal violence are therefore a key public health priority.

A detailed understanding of trends in injury numbers and injury characteristics is important to guide public health measures aimed at reducing violence^9^. Although trends in overall numbers of injuries are available from law enforcement bodies and government agencies, these data lack granularity regarding the characteristics of patients and their injuries. From a healthcare perspective, there are reports that the increase in number of stab injuries has occurred in parallel to an increase in the intensity of violence, with worsening injury severity and increases in specific injury patterns such as gluteal stab wounds^10,11^. However these concerns are based largely on anecdotal evidence, and there is a lack of detailed, contemporaneous data on injury profiles. Our previous work has shown that there are age-related differences in the timing and geographical location of stab injuries^12^, but the relationship between age and injury characteristics is poorly defined. Determining whether specific patterns of injury are more prevalent among young people as compared to their older counterparts could be used to inform educational and outreach interventions^13^.

The aim of this study was to describe contemporary injury patterns resulting from knife violence in an urban population, and examine how these injuries have changed over time. We hypothesised that there are characteristic anatomical injury patterns among young people. We also hypothesised that the number and severity of stab injuries presenting to our institution have progressively increased in recent years, and that these trends primarily involve young people rather than older age groups.

## Methods

### Study design

We performed a retrospective cohort study of all patients presenting to the trauma service at a single major trauma centre in London, UK. We screened all patients who met criteria for trauma team activation between January 2012 and December 2018 for inclusion. No major changes to the structure of our trauma system occurred during this time period. We included patients who had sustained stab injuries in an alleged assault. We excluded patients with incomplete data on age or injury characteristics, and patients whose injuries were sustained accidentally or as a result of deliberate self-harm. Demographic data, injury characteristics and clinical outcomes were collected prospectively by a dedicated data manager and stored in accordance with local information governance procedures. The study was approved by the South Central – Berkshire B National Health Service research ethics committee (reference 15/SC/0547) and was exempted from informed consent requirements owing to its retrospective design. All methods were performed in accordance with the latest revision of the Declaration of Helsinki (2013)^14^.

### Definitions

To examine age-related differences in injury patterns, we subdivided the cohort into two groups by age using the WHO definition of adolescence and young adulthood (age < 25 years)^15^. To investigate young people in more detail, we also performed subgroup analyses of injury profiles in children (age < 16), late adolescents (age 16–19) and young adults (age 20–24), as previously described^12^. Injury severity was described using abbreviated injury scores (AIS) for each anatomical region with a composite injury severity score (ISS) calculated from the square of the highest three AIS scores. Higher scores indicate a greater injury burden, with an ISS > 15 generally used to define severe trauma^16^. We defined multiple injuries as those with penetrating wounds in more than one body region. Junctional injuries were defined as those involving the groin, buttock or perineum in the lower extremity, and the axilla or shoulder in the upper extremity.

### Data analysis

Data were analysed using Microsoft Excel v16 (Microsoft, California, USA) and Prism v8.0 (GraphPad, California, USA). Additional images were generated using Tableau desktop v2020.4 (Tableau software, Washington, USA). The distribution of continuous data was assessed using quantile–quantile plots; all data were non-parametric and are reported as median with interquartile range (IQR) and were compared with Mann–Whitney U-tests or Kruskal–Wallis tests. Categorical data are displayed as number and percentage and were compared with Fisher's exact test or Chi-squared tests. Linear regression models were used to examine trends over time. Average annual changes in number of injuries were calculated from the slope of the regression line and reported with 95% confidence intervals (CI). A two-tailed *p* value less than 0.05 was considered significant throughout.

## Results

During the seven-year study period, a total of 21,231 adult trauma team activations were recorded, of whom 4,248 had sustained stab injuries. Of these, 199 had insufficient data and 466 had injuries due to DSH or accidents, leaving 3,583 patients in the final analysis. The majority of these patients (56%, 2005/3583) were young people (age < 25 years), with a median age of 19 years (IQR 17–22) in this group; in the older group (age ≥ 25 years) the median age was 32 (IQR 28–41). Females accounted for 4% of the cohort (158/3583) and had a higher median age compared to males (31 (23–40) vs 23 (19–30), *p* < 0.001) but similar injury severity scores (1 (1–9) vs 1 (1–9), *p* = 0.67). Overall, stab injuries resulting from assault accounted for 17% of all trauma team activations. Among young people, this proportion was significantly higher as compared to adults aged 25 and over (30% vs 11%, *p* < 0.001).

**Figure 1:**
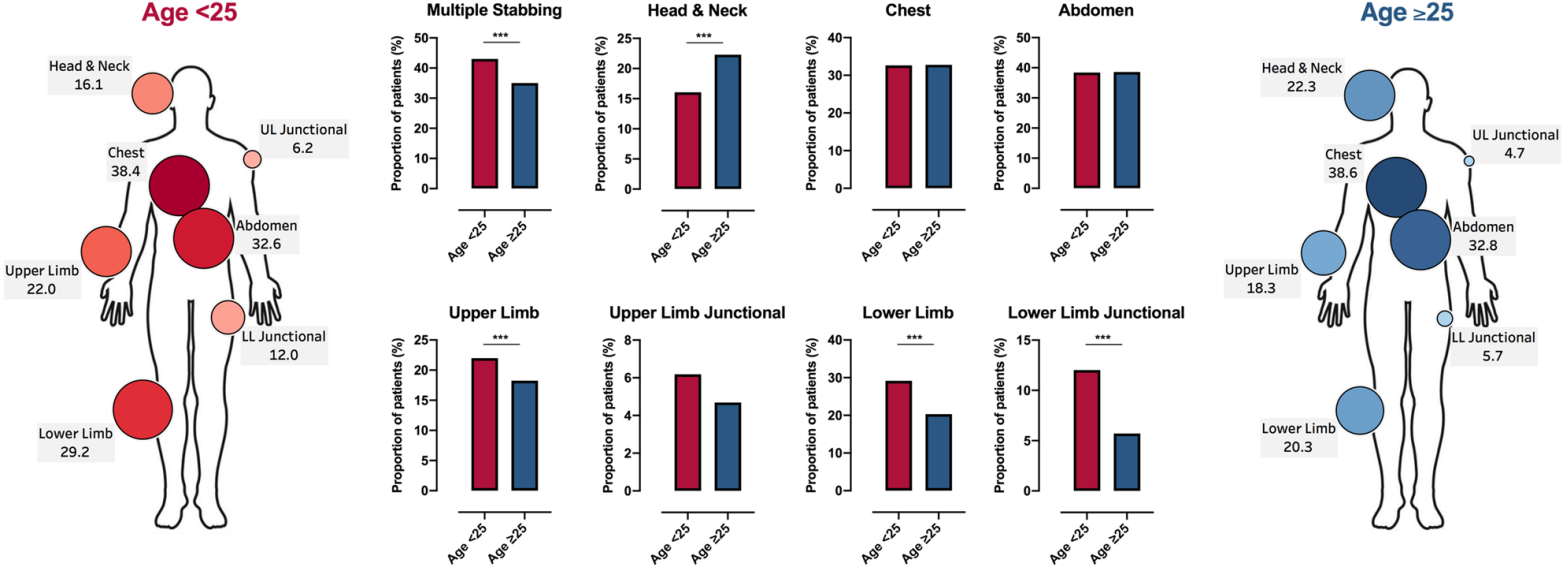
Age-related variation in anatomical profiles of stab injury. Circle size and colour in heatmaps depict proportion of patients with an injury in each area. Values are percentage of patients with an injury in the stated area. ***p < 0.001, Fisher’s exact test. *LL* lower limb, *UL* upper limb.

There were several differences in anatomical injury profiles among young people compared to older patients (Fig. 1). Injuries to multiple body regions occurred more frequently in young people (43% vs 35%, *p* < 0.001), although overall injury severity scores were similar (young people: 1 (1–9) vs older people: 1 (1–9), *p* = 0.30). In young people, stab wounds to the upper and lower limbs accounted for a significantly higher proportion of injuries, whereas there were no differences in the proportions of torso wounds and a higher frequency of wounds to the head, neck and face among older people (Fig. 1). Junctional injuries involving the lower extremity were twice as frequent in young people (12% vs 6%, *p* < 0.001), but there were no differences in upper extremity junctional injury rates (6% vs 6%, *p* = 0.51). Compared to the older age group, young people had comparable rates of surgical intervention (31% vs 30%, *p* = 0.92), major haemorrhage protocol activation (8% vs 8%, *p* = 0.53) and mortality (2% vs 2%, *p* = 0.22).

Among those aged under 25, lower limb injuries were more prevalent in children (65/191, 34%) and late adolescents (284/895, 32%) compared to young adults (236/919, 26%, *p* = 0.005). There were no other major differences in injury characteristics, intervention rates or outcomes between these age groups, although there was a trend towards higher rates of both multiple and major injuries in those aged 20–24 compared to the younger groups (Supplemental table 1).

**Figure 2:**
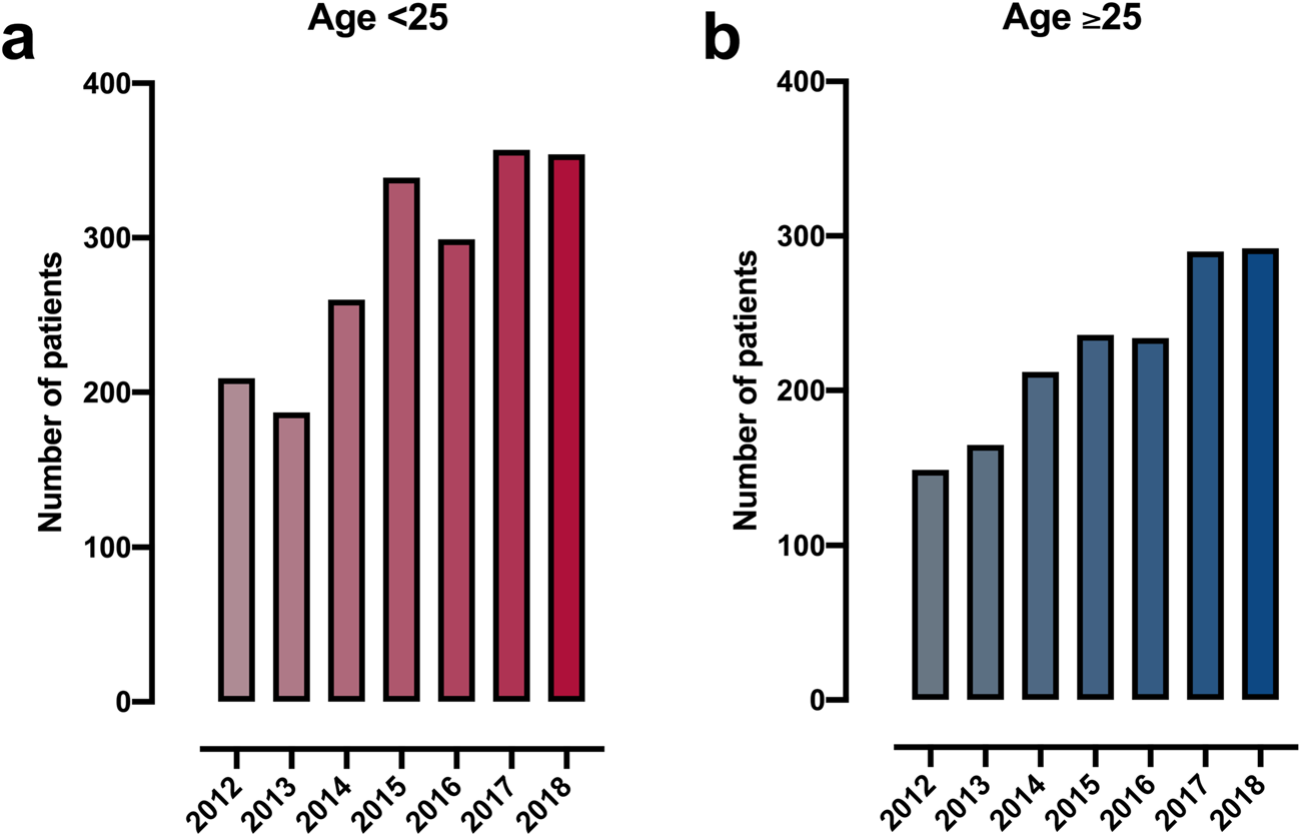
Annual number of stab injuries in young people (age under 25 years, **A**) and older people (age 25 years and over, **B**).

We next evaluated trends in number and characteristics of stab injuries over time. The annual number of stabbings increased steadily during the study period in both young people (r^2^ = 0.82, *p* = 0.005) and the over 25 age group (r^2^ = 0.95, *p* < 0.001; Fig. 2A, B). This increase equated to an average of 54 (95% CI 38–72) additional stabbings each year, of which 29 (95% CI 13–45) were in young people and 25 (95% CI 18–32) were in the older group. We found a similar pattern in children, adolescents and young adults, with a significant increase in injury numbers in all three subgroups (Supplemental figure 1). There were no significant changes over time in either the median age (r^2^ = 0.06, *p* = 0.59), the proportion of the cohort aged under 25 (r^2^ = 0.04, *p* = 0.26), or in the proportion of female patients (r^2^ = 0.46, *p* = 0.09).

**Figure 3:**
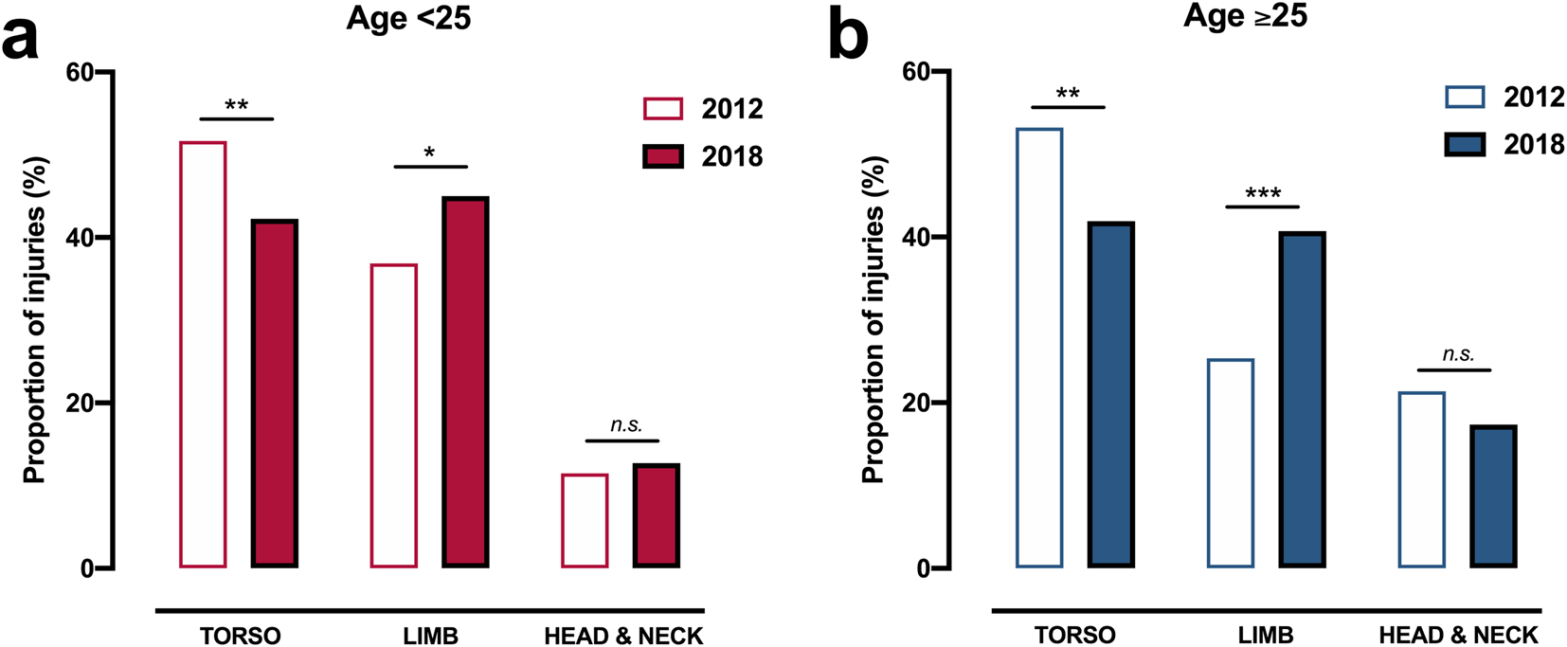
Changes in injury characteristics over time. (**A**) Young people (age < 25). (**B**) Older people (age ≥ 25). *p < 0.05; **p < 0.01; ***p < 0.001, *n.s.* not significant, Fisher’s exact test.

Finally, we examined how injury characteristics changed over the 7-year study period. In young people, stabbings to the limbs and junctional regions accounted for an increasing proportion of injuries, whereas torso wounds became less frequent over time and the proportion of head and neck injuries did not change (Fig. 3A). A similar pattern was evident in those aged over 25 (Fig. 3B). However, there were no significant temporal changes in the proportion of patients requiring surgical intervention (age < 25: r^2^ = 0.49, *p* = 0.12 ; age ≥ 25: r^2^ = 0.56, *p* = 0.09) or activation of the major haemorrhage protocol (age < 25: r^2^ = 0.37, *p* = 0.20; age ≥ 25: r^2^ = 0.06, *p* = 0.63), nor was there any significant trend in mortality rates over time (age < 25: r^2^ = 0.17, *p* = 0.70; age ≥ 25: r^2^ = 0.01, *p* = 0.84).

## Discussion

This cohort study of more than 3,500 patients describes the characteristics and recent trends in stab injury among an urban population. We found a particularly high level of knife violence among young people, in whom stabbings account for 1 in 3 trauma team activations at our institution. The anatomical profile of injuries among young people is different to their older counterparts, with a higher rate of multi-region stabbings and more frequent injuries to the groin, buttocks and limbs. Over recent years, the number of stabbings presenting to our institution has steadily increased in all age groups, accompanied by a shift in injury characteristics towards an increasing proportion of stab wounds to the limbs and junctional regions.

These results demonstrate a sustained high level of interpersonal violence involving knives over several years, with a progressive increase in caseload that has so far proved resistant to efforts at prevention. This reflects recent trends in our population, with consistent annual increases in the number of violent offences over the past decade^17^. Knife violence can therefore be most accurately be described as endemic in our community, rather than as an ‘epidemic’. Co-ordinated, multi-agency initiatives have been shown to reduce rates of knife violence in several settings, with notable examples including the Glasgow violence reduction unit in Scotland^18^ and the Medellin project in Columbia^19^. These violence reduction programmes are characterized by a broad-based approach which aims to ‘rescue’ those at immediate risk of injury, largely through educational and outreach initiatives, whilst also preventing the wider population from entering the high-risk group^20,21^. This latter approach often involves early years interventions that aim to improve the overall social and psychological wellbeing of affected communities, rather than solely relying on law enforcement to punish offenders and deter weapon carriage^22,23^. Our data suggest that existing preventative measures in our population have yet to make significant inroads into the levels of violence.

There is growing consensus that interpersonal violence must be treated as a public health problem in order to achieve significant reductions in injury rates^24,25^. The public health approach to violence reduction is advocated by several major institutions including the Centre for Disease Control and Prevention (CDC) and the World Health Organization^26^, and includes four components—defining the problem; identifying risk and protective factors; developing and testing prevention strategies; and ensuring widespread implementation. Our study adds to the first two components of this approach by defining the scale and characteristics of stab injury in our population and describing age-related risk factors.

We found that certain injury patterns are more frequent in young people, specifically junctional and extremity wounds. We also observed that the anatomical profiles of stab injuries have changed over time, with a disproportionately large increase in limb injuries during our study period that was particularly evident among young people. Bystander management of these wounds is a particular focus of community-based initiatives such as ‘YourStance’ in the UK and ‘Stop the Bleed’ internationally, which emphasise the importance of early wound compression to control extremity haemorrhage as well as other trauma-specific basic first aid measures^27,28^. Given that patients with extremity and junctional wounds are most likely to benefit from these interventions, our data support policies to further expand these initiatives in at-risk communities.

Although the anatomical profile of injuries has changed over time, we did not find evidence of an increase in the proportion of patients with severe or fatal injuries during our study period. Instead we found global increases in all subtypes of injury in all of the age groups we studied, including both major and minor injuries in children, adolescents and young adults. We believe this data suggests increasing normalisation of knife possession and utilisation of knives in confrontations, reflecting an increase in the number of individuals at risk of injury, rather than an increase in knife violence associated with gang activities and organised crime. This interpretation would again support expanded use of educational and outreach initiatives to raise awareness of the implications of knife violence and reduce weapon carriage. These strategies are central pillars of effective violence reduction policies and have been shown to be effective in other settings^18,19^. It must be emphasised that although the majority of the rise in case numbers in our study were injuries categorised as low injury severity, even physically minor injuries sustained in assaults can have major impact on the psychological and social wellbeing of the victim^5–7^. This is especially true among children and adolescents, in whom exposure to violence is strongly associated with risk of subsequent behavioural problems, suicide and involvement in further violent incidents^7,8^.

Another important observation in this study is that a substantial proportion of stab injuries presenting to our institution are from an older age group, and that the annual numbers of stabbings in this population has also increased markedly over recent years. Given that young people are at highest risk, the majority of violence reduction initiatives and media coverage is focused on stabbings in this age group. However, our study illustrates that interpersonal violence involving knives is also a significant problem in older ages, accounting for 10% of all trauma team activations at our institution in those aged over 25. This older group is clearly heterogeneous in terms of epidemiology and risk factors for injury, and further study to characterise this population and inform efforts at prevention Is warranted.

There are several limitations to this study. First, as a single-centre study the generalisability of our findings to other populations is unclear. Second, we did not have access to population level data and therefore we were not able to adjust for changes in size of our catchment population or calculate absolute risk rates. Third, although well-established, the cut-offs we used to compare age-related differences in injury patterns are arbitrary and may mask unrecognised within-group heterogeneity given the wide age range of the study cohort. Fourth, we did not perform a detailed analysis of the impact of gender on stab injury trends as this is beyond the scope of our study and had been investigated in detail previously^29^; gender may have a confounding effect, although because of the small number of females in this cohort this is unlikely to alter the overall conclusions. However, females in our cohort had a higher median age and may have different injury characteristics, and therefore further study in this area is important. Finally, a longer time period of study would enable more detailed assessment of trends over time and would have greater potential to identify long-term impacts of preventative initiatives on injury rates.

In summary, this study provides a detailed insight into the characteristics of stab injuries presenting to an urban major trauma centre. Our central observation is that knife violence remains a major public health problem in our community, with escalating numbers of injuries in both young and older people. There are age-related differences in injury profiles which support the use of targeted educational and outreach initiatives in high-risk groups, particularly emphasising the risks of weapon carriage and focusing on control of extremity haemorrhage. Our results support expansion of these programmes in at-risk communities, integrated within a coordinated public health approach that also encompasses long-term, broad-based preventative initiatives to deliver sustained reductions in interpersonal violence.

## Supplementary Information

Supplementary Information.
