# Supplementary material for: Age-related injury patterns resulting from knife violence in an urban population
Source: Sci Rep. 2022 Sep 26;12:15250. doi: 10.1038/s41598-022-17768-x (PMC9512781; doi:10.1038/s41598-022-17768-x)
Supplement: Supplementary file 1 — Supplementary Information. [file 41598_2022_17768_MOESM1_ESM.pdf]

SUPPLEMENTAL MATERIAL

Manuscript Title: Age-related Injury Patterns Resulting from Knife Violence in an Urban Population

Authors: Vulliamy P[1], Hancorn K[2], Glasgow S[1], West A[2], Davenport RA[1,2], Brohi K[1,2], Griffiths MP[2]*

[1] Centre for Trauma Sciences, Blizard Institute, Queen Mary University of London, E1 2AT
[2] The Royal London Hospital Major Trauma Centre, Barts Health NHS Trust, Whitechapel, E1 1FR
*Corresponding author. Email: Martinpatrick.griffiths@nhs.net

Supplemental Figure 1

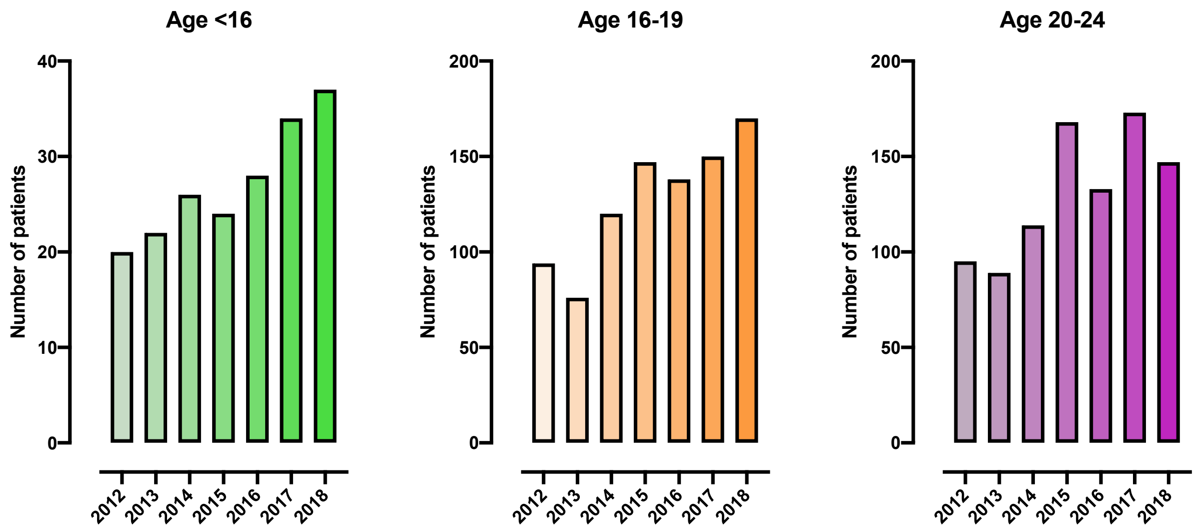

Supplemental Figure 1: Annual number of patients with stab injuries in children (Aged <16), adolescents (age 16-19) and young adults (age 20-24).

Supplemental Table 1

Number of Patients	Age <16<br>191	Age 16-19<br>895	Age 20-24<br>919	p-value
Patient characteristics				
Age, years[1]	15 (14-15)	18 (17-18)	22 (21-23)	**<0.001**
Male	185 (97)	884 (99)	893 (97)	**0.04**
Injury Characteristics				
Injury Severity Score[1]	1 (1-5)	1 (1-9)	1 (1-9)	0.49
ISS>9	27 (14)	147 (16)	178 (19)	0.11
ISS>15	19 (10)	85 (9)	95 (10)	0.84
Multiple injuries	118 (38)	523 (42)	506 (45)	0.14
Head/Neck/Face	26 (14)	131 (15)	165 (18)	0.10
Chest	66 (35)	335 (37)	369 (40)	0.25
Abdomen	61 (32)	296 (33)	297 (32)	0.92
Upper Limb	36 (19)	182 (20)	223 (24)	0.07
Lower limb	65 (34)	284 (32)	236 (26)	**0.005**
Upper Limb Junctional	8 (6)	54 (6)	62 (7)	0.40
Lower Limb Junctional	24 (13)	117 (13)	100 (11)	0.35
Major Haemorrhage Protocol	11 (6)	79 (9)	68 (7)	0.28
Outcomes				
Discharge home from ED	53 (28)	328 (37)	339 (37)	**0.046**
Surgical procedure	67 (35)	269 (30)	291 (32)	0.37
In-hospital mortality	1 (1)	14 (2)	18 (2)	0.35

Supplemental Table 1: Characteristics of children (age <16), adolescents (age 16-19) and young adults (age 20-24). Values are n (%) unless stated. P-values report three groups comparisons derived from Kruskal-Wallis test for continuous variables and chi-squared test for categorical variables. [1]values are median (interquartile range). ISS, Injury Severity Score. ED, Emergency Department.
